# The Impact of Screening for Perioperative ICU Admission in Geriatric Hip Fracture Patients: A Retrospective Analysis

**DOI:** 10.7759/cureus.49234

**Published:** 2023-11-22

**Authors:** Charles Fasanya, John J Lee, Catherine G Caronia, Lauren Rothburd, Tenzing Japhe, Young Hee Hahn, Dajana Reci, Patricia Eckardt

**Affiliations:** 1 Trauma, Good Samaritan University Hospital, West Islip, USA; 2 Orthopaedic Surgery, Good Samaritan University Hospital, West Islip, USA; 3 Pediatrics, Good Samaritan University Hospital, West Islip, USA; 4 Trauma, New York Institute of Technology, West Islip, USA; 5 Nursing, Good Samaritan University Hospital, West Islip, USA

**Keywords:** risk factors, propensity score, hip fractures, intensive care unit, screening

## Abstract

Background: Hip fracture patients are a subset of trauma patients with high peri-operative mortality. To mitigate the mortality risk, the use of predictive scoring systems (e.g., RSI or Nomograms) for risk stratification and monitoring of high-risk patients in the intensive care unit (ICU) has been proposed. Screening patients for ICU admission with relatively low-cost tools may achieve high-quality, low-cost care. The aim of this study was to assess the effectiveness and feasibility of screening postoperative hip fracture patients for ICU admission.

Methods: This is a retrospective single-site study comparing two groups of patients, before and after implementation of a hip fracture postoperative screening intervention in a level 1 trauma center in the United States. All hip fracture patients > 55 years of age admitted to the hospital between January 2021 and May 2023 were included. Trauma team members assessed and screened patients postoperatively in the post-anesthesia care unit (PACU), ordering standardized tests, including laboratory tests, a chest x-ray, and electrocardiogram (EKG). Assessment of the effect of the intervention included a comparison of a number of major adverse events (MAEs), mortality, planned and unplanned ICU admissions, ICU length of stay (LOS), and hospital LOS between pre- and post-intervention groups. Propensity score (PS) estimates were used to compare outcomes between the matched participants in the sample. A predictive model for ICU admission for the overall sample was estimated, and discriminative ability was assessed with an area under the curve (AUC) receiver operator characteristics (ROC) analysis. Lastly, feasibility was assessed by compliance with screening intervention and charges per patient related to the intervention.

Results: The sample consisted of 290 patients in the pre-intervention and 180 patients in the post-intervention groups, respectively, with a mean age of 81.4 ± (9.9) years. There was a significant increase (p<0.01) in planned ICU admissions (OR=2.387, 95% CI (1.430, 3.983)) after screening protocol implementation. There was no significant difference between the pre-intervention group and post-intervention group in the number of MAEs (p=0.392), mortality (p=0.591), ICU LOS (p=0.617), and hospital LOS (p=0.151). When the PS-matched sample (n=424) was analyzed, there was a significant decrease (p=0.45) in unplanned ICU admissions (OR=6.40, 95% CI (0.81, 50.95)) after protocol implementation. Anticoagulants, emergency department (ED) respiratory rate (RR), injury severity score (ISS), number of comorbidities, substance use disorder (SAD), peripheral artery disease (PAD), and chronic obstructive pulmonary disease (COPD) were significant predictors of ICU admission (p=0.002, 0.022, 0.030, 0.034, 0.039, 0.039, and 0.042), respectively, and, demonstrated the discriminative ability between high and low risk for ICU admission (AUC=0.597, 0.587, 0.581, 0.578, 0.513, and 0.587, respectively). The screening intervention was achievable with 99% compliance (Kappa estimate 0.94) among trauma team members with an average charge of $282 per patient.

Conclusion: The addition of a postoperative screening intervention for hip fracture patients > 55 years of age is achievable and decreases unplanned ICU admissions in matched samples. Presenting clinical indicators and comorbidities are associated with ICU admission and provide sufficient discriminatory ability as criteria for ICU admission.

## Introduction

The incidence of hip fractures in the United States has been estimated at approximately 250,000 cases annually [1,2], with geriatric hip fractures, in particular, causing significant morbidity and mortality [3-6]. It is well-known in the field that one-year post-fracture mortality can be as high as 30% [7,8], and although these rates have decreased over the years, recent statistics still report a substantial 22% one-year mortality rate in hip fracture patients [9]. A National Trauma Databank study from 2014 retrospectively reviewed 44,419 hip fracture patients and found an in-hospital mortality of 4.5% [10]. To further complicate matters, there are few standardized protocols for determining the appropriate perioperative care required to reduce mortality in hip fracture patients [11]. Ideally, all high-risk hip fracture patients would be admitted for ICU observation postoperatively. However, due to limited resources, particularly in busy level 1 trauma centers, there is not always ample capacity in the surgical intensive care unit (SICU). Additionally, not all hip fracture patients require an intensive care unit (ICU) level of care, further highlighting the need for optimized perioperative screening and intervention.

Perioperative comprehensive geriatric assessment (CGA) programs designed to address this issue have been associated with better outcomes, including decreased length of stay (LOS), improved functional status upon discharge, and decreased mortality [12]. Existing studies have expanded previously validated mortality risk scoring systems, such as the Score for Trauma Triage in the Geriatric and Middle-Aged (STTGMA), in order to improve its predictions of mortality, ICU LOS, and overall risk stratification [13,14]. Other groups have used a risk stratification index (RSI) to facilitate ICU resource utilization post-hip fracture surgery [15], or the Charlson Comorbidity Index (CCI) to improve predictions of postoperative complications after geriatric hip fracture surgery [16]. N-terminal pro-B-type natriuretic peptide (NT-pro-BNP)/brain natriuretic peptide (BNP) in various studies has been used to screen high-risk patients preoperatively for elective non-cardiac and hip fracture surgery [17,18]. Troponin has also been shown to be predictive of short-term and long-term mortality in hip fracture patients [18]. The electrocardiogram (EKG) has not been shown to be helpful in predicting mortality unless ST-segment elevation is present [18]. Ultimately, the resources to provide ICU care to all hip fracture patients, in addition to other cohorts of patients, can be prohibitive for trauma centers with a limited number of critical care beds. Additionally, only a few studies in the field have developed trigger algorithms specifically to determine which hip fracture patients should be admitted to the ICU perioperatively [15]. Targeted approaches using an initial assessment and a postoperative trigger algorithm can be successful in improving outcomes within a resource-constrained setting.

In our institution, hip fracture patients are admitted to the trauma service and managed by trauma and orthopedic surgeons. Preoperatively, high-risk patients require subspecialist evaluation (typically cardiac or pulmonary) for risk assessment and stratification. However, postoperatively, a standardized screening assessment was not utilized to assess patients who, during the preoperative period, were determined not to be high risk to require ICU admission. Therefore, to determine if patients requiring critical care resources could better be identified to improve resource utilization, a postoperative screening protocol was designed and implemented. The screening was completed in the PACU within 90 minutes of a patient’s arrival at the PACU. This study was completed to evaluate whether screening of patients, in conjunction with an established geriatric hip fracture protocol, would reduce postoperative complications. In addition to the clinical assessment of patients, relatively inexpensive screening tests were utilized in the evaluation and include plain radiographs (chest X-ray), EKG, and laboratory values, including complete blood count (CBC), basic metabolic panel (BMP), lactic acid, troponin, and BNP.

The objective of this study was to assess the feasibility and effectiveness of an innovative and easily reproducible postoperative hip fracture patient assessment protocol to determine the need for critical care to reduce perioperative adverse events and mortality.

## Materials and methods

This was an investigator-initiated single-institution retrospective cohort study of all hip fracture patients (> 55 years of age) admitted to our institution over a one-year period, six months prior to implementing an interventional protocol and six months following its implementation (January 1, 2022, and December 31, 2022). After approval from the institutional review board for research with human subjects (Good Samaritan University Hospital Office, IRB#: 2023.05.17.10), the data were abstracted from the National Trauma Data Registry and patients’ electronic medical records (EMR). The inclusion and exclusion criteria were the same for the pre-intervention and post-intervention groups. Inclusion criteria were hip fracture patients > 55 years of age admitted to the trauma center six months prior to the protocol initiation and six months after. Exclusion criteria were patients = < 55 years of age, patients treated with non-operative management of hip fractures, and patients admitted to hospice.

Statistical analyses

The analysis was completed using Statistical Product and Service Solutions (SPSS) for Windows, version 28 (IBM Corp., Armonk, N.Y., USA) and Stata Statistical Software: Release 18 (StataCorp LLC, College Station, Texas, USA). The reporting of studies conducted using the observational routinely collected health data (RECORD) guidelines were used for reporting study results. Independent sample t-tests were used to compare the means between two groups (pre- and post-intervention samples) on the continuous variables. Chi-square (χ2) tests were used to compare the proportional differences between the two groups (pre- and post-intervention samples) on the nominal variables. Fischer’s exact test (FET) was conducted to analyze categorical data if more than 20% of cells had expected counts less than 5. Inferential analyses used a two-tailed approach, and 0.05 was chosen as the a priori critical alpha. The effect of the intervention was estimated by comparison of the number of major adverse events (MAEs), mortality, planned and unplanned ICU admissions, ICU LOS, and hospital LOS between pre- and post-intervention groups. Additionally, to increase the validity of treatment effect estimates, propensity scores (PS) were employed to match patients within quintiles on variables identified in the literature and within this sample associated with grouping variable (pre- or post-intervention) or the outcomes of interest (mortality, ICU admission, ICU and hospital LOS). The variables in the PS estimates included CCI, orthopedic frailty score (OFS), age, falls, anticoagulation status, initial emergency department (ED), respiratory rate (RR), increased initial injury severity score (ISS), number of comorbidities, substance use disorder (SAD), peripheral artery disease (PAD), and chronic obstructive pulmonary disease (COPD). Patients in the PS-matched sample were balanced on all covariates prior to inferential estimates. Individual variables found to be predictive of ICU admission in the overall model were assessed for discriminative ability using the area under the curve (AUC) analysis from the receiver operating characteristic (ROC) curve. Lastly, feasibility was assessed as provider compliance with screening intervention and charges per patient related to the intervention.

## Results

Sample and sample characteristics

There was a total of 518 patients in the study between January 1, 2022, and December 31, 2022. Of these, 48 patients treated non-operatively met exclusion criteria, of which six died. The final sample comprised 470 patients (n=290 and n=180) in the pre- and post-intervention groups, respectively, with a mean age of 81.4 ± (9.9) years. The mean age of participants was 80 and 82, respectively, for pre- and post-intervention groups (Table 1). Over 40% of the sample were over 84 years of age in both groups. Over 70% of both groups were female. The majority of the mechanism of injury (MOI) was falls (98% and 95%). ISS was expectedly low at approximately 9.5 for both groups. The Glasgow coma score (GCS) averaged just below 15. Medicare was the primary insurance payor for 85-90% of patients. Overall, the comorbidities between the pre- and post-intervention groups were comparable (Table 2). CCI and OFS, which are a composite of risk factors for morbidity and mortality, were not significantly different between patients pre- and post-intervention. However, the percentage of patients with COPD, dementia (p=0.01), or SAD (p=0.02) was significantly higher in the post-intervention group (p=0.002, p=0.01, p=0.02, respectively). Patients with advance directives limiting care in place and with a history of psychiatric disorders were significantly higher for the pre-intervention group (p=0.008, p=0.006, respectively).

**Table 1:** Demographics of the study sample Abbreviations: SD, standard deviation; n, sample size; MOI, mechanism of injury; ED, emergency department; SBP, systolic blood pressure; HR, heart rate; RR, respiratory rate; ---, not applicable.

Variables	Pre-intervention (n=290)	Post-intervention (n=180)	P value	OR (95% CI)
Age, mean ± SD	80.5 ± 10.0	82.3 ± 9.1	0.048	---
Over age 84 n (%)	120 (41.5)	86 (48.6)	0.136	1.33 (0.91, 1.93)
Gender				
Male n (%)	74 (25.5)	48 (26.7)	0.782	1.06 (0.69, 1.63)
Female n (%)	216 (74.5)	132 (73.3)	0.782	0.95 (0.62 1.43)
Mechanism of injury				
Fall n (%)	284 (97.9)	171 (95.0)	0.079	0.40 (0.14, 1.14)
Motor Vehicle Traffic n (%)	3 (1.0)	4 (2.0)	0.301	2.17 (0.48, 9.82)
Other MOI n (%)	3 (1.0)	5 (2.8)	0.156	2.73 (0.64, 11.57)
Glasgow Coma Scale, mean ± SD	14.8 ± 0.6	14.7 ± 1.1	0.479	---
Injury Severity Score, mean ± SD	9.5 ± 1.8	9.6± 1.7	0.537	---
Insurance				
Medicare n (%)	248 (85.5)	163 (90.6)	0.109	1.62 (0.89, 2.95)
Private Insurance n (%)	22 (7.6)	7 (3.9)	0.105	0.49 (0.20, 1.17)
Other Insurance n (%)	20 (6.9)	10 (5.6)	0.563	0.79 (0.36, 1.73)
Revised Trauma Score (RTS)	7.8 ± 0.3	7.8 ± 0.3	0.699	(-0.06, 0.04)
Initial ED SBP (mmHg)	144.3 ± 25.5	147.5 ± 26.7	0.184	(-7.84, 1.50)
Initial ED HR (bpm)	80.4 ± 15.4	79.6 ± 15.7	0.598	(-2.04, 3.54)
Initial ED RR, breaths per minute	18.4 ± 2.2	18.7 ± 2.5	0.228	(-0.68, 0.16)
Initial ED O2 Saturation	96.9 ± 2.5	96.5 ± 3.1	0.113	(-0.09, 0.89)
HCT	36.9 ± 5.4	36.99 ± 5.8	0.944	(-1.07, 1.00)

**Table 2:** Comorbidities of the samples pre- and post-intervention Abbreviations: SD, standard deviation; n, sample size; ADD, attention-deficit disorder; ADHD, attention-deficit hyperactivity disorder; COPD, chronic obstructive pulmonary disease; ---, not applicable.

Variables	Pre-intervention (n=290)	Post-intervention (n=180)	P value	OR (95% CI)
Charleston Comorbidity Index, mean ± SD	0.8 ± 1.1	0.9 ± 1.1	0.213	---
Orthopedic Fragility Index, mean ± SD	1.1 ± 0.8	1.2 ± 0.9	0.177	---
Co-Morbidities, mean ± SD	2.5 ± 1.5	2.7 ± 1.4	0.234	---
ADD/ADHD n (%)	1 (0.3)	0 (0.0)	0.430	---
Advanced Directive Limiting Care n (%)	11 (3.8)	0 (0.0)	0.008	---
Alcohol Use Disorder n (%)	10 (3.4)	11 (6.1)	0.174	1.82 (0.75, 4.38)
Angina Pectoris n (%)	1 (0.3)	0 (0.0)	0.430	---
Anticoagulant n (%)	78 (26.9)	50 (27.8)	0.835	1.04 (0.68, 1.58)
Bipolar n (%)	0 (0.0)	0 (0.0)	---	---
Cerebrovascular Accident n (%)	9 (3.1)	8 (4.4)	0.449	1.45 (0.55, 3.83)
Chemotherapy n (%)	3 (1.0)	1 (0.6)	0.583	0.53 (0.05, 5.17)
Cirrhosis n (%)	2 (0.7)	1 (0.6)	0.859	0.80 (0.07, 8.93)
Congestive Heart Failure n (%)	27 (9.3)	16 (8.9)	0.878	0.95 (0.49, 1.81)
COPD n (%)	26 (9.0)	34 (18.9)	0.002	2.365 (1.36, 4.09)
COVID-19 n (%)	19 (6.6)	11 (6.1)	0.849	0.92 (0.43, 1.99)
Dementia n (%)	54 (18.6)	52 (28.9)	0.010	1.77 (1.14, 2.75)
Dependent Health n (%)	154 (53.1)	103 (57.2)	0.383	1.18 (0.81, 1.71)
Diabetes Mell n (%)	57 (19.7)	29 (16.1)	0.334	0.785(0.48, 1.28)
Dysplasia n (%)	4 (1.4)	3 (1.7)	0.803	1.21 (0.26, 5.47)
Hypertension n (%)	216 (74.5)	135 (75.0)	0.900	1.02(0.67, 1.57)
Mentality Personality n (%)	19 (6.6)	2 (1.1)	0.006	0.16 (0.03, 0.69)
Myocardial infarction n (%)	0 (0.0)	2 (1.1)	0.072	---
Other Mental Disorders n (%)	0 (0.0)	1 (0.6)	0.204	---
Peripheral Arterial Disease n (%)	12 (4.1)	5 (2.8)	0.443	0.66 (0.22, 1.91)
Renal Failure n (%)	6 (2.1)	0 (0.0)	0.052	---
Smoker n (%)	23 (7.9)	15 (8.3)	0.876	1.05 (0.53, 2.08)
Steroid Use n (%)	4 (1.4)	3 (1.7)	0.803	1.21(0.26, 5.47)
Substance Abuse Disorder n (%)	1 (0.3)	5 (2.8)	0.022	8.25 (0.95, 71.25)

Outcomes of interest

**Figure 1:**
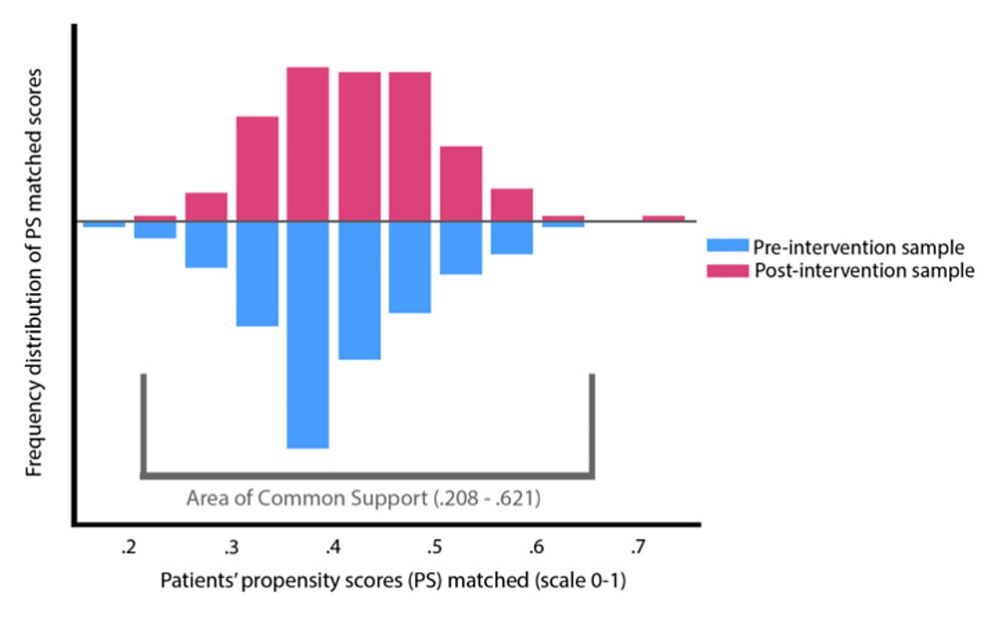
Propensity score-matched sample (n=424)

**Figure 2:**
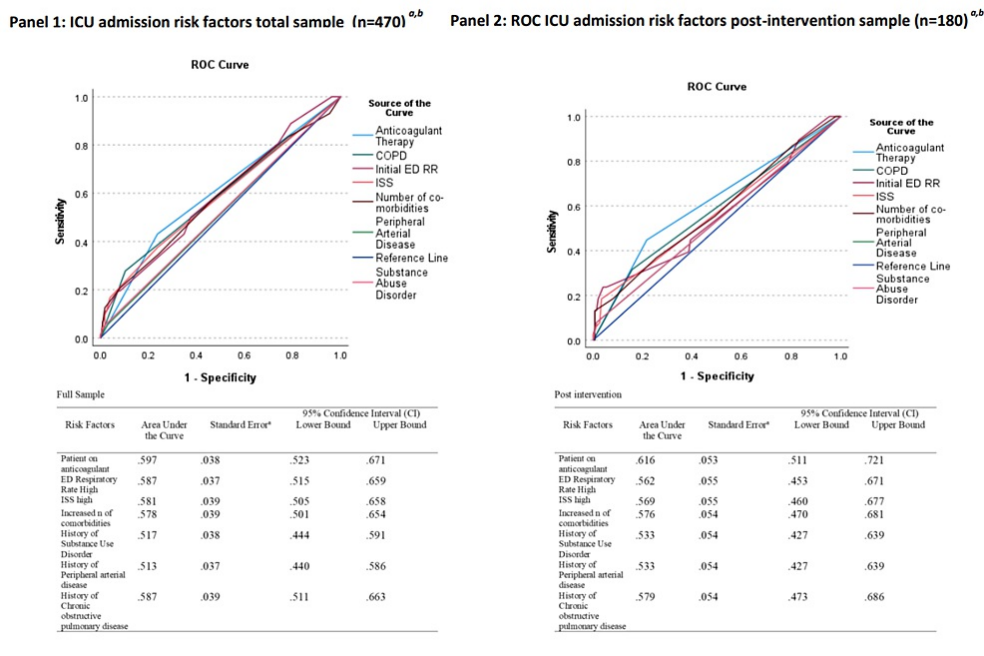
Receiver operating characteristic (ROC) curves of ICU admission ^a ^Risk factors in panels 1 and 2 have a positive relationship with the probability of ICU admission. ^b ^There is at least one tie between positive and negative state groups.

There was a significant increase (p <0.01) in planned ICU admissions (OR=2.387, 95% CI (1.430, 3.983)) after the screening protocol implementation. There was no statistically significant difference between pre-intervention and post-intervention groups in the number of MAEs (p=0.392), mortality (p=0.591), ICU LOS (p=0.617), and hospital LOS (p=0.151) when the total sample was included in the analysis (Table 3). When the PS-matched sample area of common support (n=424) was analyzed (Figure 1), there was a significant decrease (p=0.45) in unplanned ICU admissions (OR=6.40, 95% CI (0.81, 50.95)) after screening protocol implementation (Table 4). The presence of advance directives limiting care in place was not included in the final PS-matched model as it was a perfect predictor of patients not being admitted to the ICU. The patients on anticoagulants, with increased initial ED, RR, increased initial ISS, increased number of comorbidities, SAD, PAD, and COPD, were found to be significant predictors of ICU admission (p= 0.002, 0.022, 0.030, 0.034, 0.039, 0.039, and 0.042, respectively) (Tables 5-6). Additionally, the variables found significant for ICU admission in the overall sample were found to have discriminative ability (AUC=0.597, 0.587, 0.581, 0.578, 0.513, and 0.587, respectively) and were also significant in the post-intervention sample (Figure 2). The screening intervention was found to be feasible with 99% compliance (Kappa estimate 0.94) among trauma team members. Additionally, the average charge per patient was $282 (Table 7).

**Table 3:** Primary outcomes for sample participants pre- and post-intervention Note: Major adverse events were indicated by the number of events defined in the National Trauma Data Standard as acute kidney injury, acute respiratory distress syndrome, alcohol withdrawal syndrome, cardiac arrest with cardiopulmonary resuscitation, catheter-associated urinary tract infection, central line-associated bloodstream infection, deep surgical site infection, deep vein thrombosis, delirium, myocardial infarction, organ/space surgical site infection, osteomyelitis, pressure ulcer, pulmonary embolism, severe sepsis, stroke, superficial incisional surgical site infection, unplanned admission to the ICU, unplanned visit to the operating room, and ventilator-associated pneumonia. Abbreviations: SD, standard deviation; n, sample size; ICU, intensive care unit; LOS, length of stay; ---, not applicable. ^a^ Significance tests for continuous were the two-tailed independent samples t-test. ^b ^Significance tests for categorical variables were the cross-tabulation two-tailed chi-square estimate with 1 degree of freedom (Fisher’s exact estimate for cells with expected value 5 or less).

Variables	Pre-intervention (n=290)	Post-intervention (n=180)	P value	OR (95% CI)
Major Adverse Events, mean ± SD^a^	0.27 ± 0.6	0.23 ± 0.5	0.392	---
Mortality n (%)^b^	7 (2.4)	3 (1.7)	0.591	0.68 (0.17, 2.70)
Admitted to ICU n (%)^b^	31 (10.7)	40 (22.2)	0.001	2.38 (1.43, 3.98)
Unplanned Admitted to ICU n (%)^b^	10 (3.1)	10 (5.1)	0.253	2.38 (1.43, 3.98)
ICU length of stay (days), mean ± SD^a^	5.26 ± 3.7	4.75 ± 4.6	0.617	---
Hospital length of stay (days), mean ± SD^a^	5.41 ± 3.1	5.86 ± 3.5	0.151	---

**Table 4:** Primary outcomes for sample participants on the area of common support after propensity score matched (n=424) Abbreviations: SD, standard deviation; n, sample size; ICU, intensive care unit; LOS, length of stay; MAE, major adverse events defined in the National Trauma Data Standard as acute kidney injury, acute respiratory distress syndrome, alcohol withdrawal syndrome, cardiac arrest with cardiopulmonary resuscitation, catheter-associated urinary tract infection, central line-associated bloodstream infection, deep surgical site infection, deep vein thrombosis, delirium, myocardial infarction, organ/space surgical site infection, osteomyelitis, pressure ulcer, pulmonary embolism, severe sepsis, stroke, superficial incisional surgical site infection, unplanned admission to the ICU, unplanned visit to the operating room, and ventilator-associated pneumonia; ---, not applicable. ^a^ Significance tests for the continuous variables were the two-tailed independent samples t-test. ^b^ Significance tests for categorical variables were the cross-tabulation two-tailed chi-square estimate with 1 degree of freedom (Fisher’s exact estimate for cells with expected value 5 or less). ^c ^Inferential statistics not estimated within quintiles as n=0 within four quintiles for the post-intervention group.

Variables	Pre-intervention	Post-intervention	P value	OR (95% CI)
Total PS-matched sample (n=424)				
Major Adverse Events, mean ± SD^a^	1.3 ± 0.6	1.4 ± 0.6	0.780	---
Mortality n (%)^b^	8 (3.2%)	2 (1.2%)	0.328	0.37 (0.77, 1.75)
Admitted to ICU n (%)^b^	29 (11.6%)	34 (19.7%)	0.021	1.87 (1.09, 3.21)
Unplanned Admitted to ICU n (%)^b,c^	9 (3.6%)	1 (0.6%)	0.045	6.4 (0.81, 50.9)
ICU length of stay (days), mean ± SD^a^	5.2 ± 3.7	4.8 ± 4.6	0.698	---
Hospital length of stay (days), mean ± SD^a^	5.4 ± 3.2	5.7 ± 3.4	0.338	---
Quintile 1 PS-matched sample (n=85)				
Major Adverse Events, mean ± SD^a^	1.3 + 0.6	1.3 + 0.7	0.954	---
Mortality n (%)^b^	3 (5.6%)	0 (0.0%)	0.548	0.94 (0.88, 1.00)
Admitted to ICU n (%)^b^	8 (14.8%)	5 (17.2%)	0.761	1.19 (0.35, 4.06)
ICU length of stay (days), mean ± SD^a^	5.2 ± 4.6	3.8 ± 1.9	0.524	---
Hospital length of stay (days), mean ± SD^a^	5.8 ± 3.6	4.7 ± 1.6	0.075	---
Quintile 2 PS-matched sample (n=85)				
Major Adverse Events, mean ± SD^a^	1.3 ± 0.6	1.1 ± 0.5	0.084	---
Mortality n (%)^b^	1 (1.8%)	0 (0.0%)	1.000	0.98 (0.94, 1.01)
Admitted to ICU n (%)^b^	5 (8.8%)	3 (10.7%)	0.773	1.24 (0.27, 5.64)
ICU length of stay (days), mean ± SD^a^	7 ± 6.3	2.3 ± 0.5	0.266	---
Hospital length of stay (days), mean ± SD^a^	5.5 ± 4	5.2 ± 1.9	0.749	---
Quintile 3 PS-matched sample (n=85)				
Major Adverse Events, mean ± SD^a^	1.2 ± 0.6	1.5 ± 0.6	0.048	---
Mortality n (%)^b^	1 (1.7%)	0 (0.0%)	0.485	0.98 (0.95, 1.01)
Admitted to ICU n (%)^b^	8 (13.8%)	10 (35.7%)	0.019	3.47 (0.17, 0.087)
ICU length of stay (days), mean ± SD^a^	4.6 ± 2.1	3.3 ± 1.8	0.182	---
Hospital length of stay (days), mean ± SD^a^	5.1 ± 2.6	5.6 ± 2.7	0.464	---
Quintile 4 PS-matched sample (n=85)				
Major Adverse Events, mean ± SD^a^	1.3 ± 0.5	1.3 ± 0.6	1.00	---
Mortality n (%)^b^	0	0	---	---
Admitted to ICU n (%)^b^	5 (11.9%)	6 (14.3%)	0.746	1.23 (0.34, 4.40)
ICU length of stay (days), mean ± SD^a^	4.8 ± 2	5.67 ± 4.84	0.719	---
Hospital length of stay (days), mean ± SD^a^	5.2 ± 2.9	6.3 ± 4.6	0.193	---
Quintile 5 PS-matched sample (n=84)				
Major Adverse Events, mean ± SD^a^	1.3 ± 0.5	1.3 ± 0.6	0.880	---
Mortality n (%)^b^	3 (7.5%)	2 (4.7%)	0.668	0.60 (0.09, 3.80)
Admitted to ICU n (%)^b^	3 (7.5%)	10 (21.7%)	0.066	3.42 (0.87, 13.47)
ICU length of stay (days), mean ± SD^a^	4.6 ± 2.3	7.1 ± 6.9	0.575	---
Hospital length of stay (days), mean ± SD^a^	5.1 ± 2.4	6.1 ± 4.2	0.204	---

**Table 5:** Post-intervention patients “at risk” for ICU admission with risk factors of age, mechanism of injury, and comorbidities Abbreviations: ICU, intensive care unit; n, sample size; MOI, mechanism of injury; COPD, chronic obstructive pulmonary disease; ADD, attention-deficit disorder; ADHD, attention-deficit hyperactivity disorder; ---, not applicable. ^a^Comorbidities listed are as defined in the National Trauma Data Standard

n (%)	Admitted to ICU (n=40)	Not Admitted to ICU (n=140)	ρ value	OR (95% CI)
Older than 84 years old	20 (50.0)	69 (49.3)	0.936	1.02 (0.51, 2.07)
CDC MOI = Fall	36 (90.0)	135 (96.4)	0.100	0.33 (0.08, 1.30)
Co-morbidities^a^:				
Alcohol Use Disorder	2 (5.0)	9 (6.4)	0.739	0.76 (0.15, 3.69)
Bleeding Disorder	0 (0.0)	0 (0.0)	---	---
Currently Receiving Chemotherapy for Cancer	0 (0.0)	1 (0.7)	0.592	---
Congenital Anomalies	0 (0.0)	0 (0.0)	---	---
Congestive Heart Failure	5 (12.5)	11 (7.9)	0.363	1.67 (0.54, 5.14)
Current Smoker	2 (5.0)	13 (9.3)	0.387	0.51 (0.11, 2.38)
Chronic Renal Failure	0 (0.0)	0 (0.0)	---	---
Cerebrovascular Accident	2 (5.0)	6 (4.3)	0.847	1.17 (0.22, 6.06)
Diabetes Mellitus	5 (12.5)	24 (17.1)	0.481	0.69 (0.24, 1.94)
Disseminated Cancer	1 (2.5)	2 (1.4)	0.641	1.76 (0.15, 20.02)
Advanced Directive Limiting Care	0 (0.0)	0 (0.0)	---	---
Functionally Dependent Health Status	18 (45.0)	85 (60.7)	0.076	0.52 (0.26, 1.07)
Hypertension	33 (82.5)	102 (72.9)	0.214	1.75 (0.71, 4.30)
Prematurity	0 (0.0)	0 (0.0)	---	---
COPD	12 (30.0)	22 (15.7)	0.042	2.29 (1.01, 5.19)
Steroid Use	1 (2.5)	2 (1.4)	0.641	1.76 (0.15, 20.02)
Cirrhosis	1 (2.5)	0 (0.0)	0.061	---
Dementia	15 (37.5)	37 (26.4)	0.173	1.67 (0.79, 3.50)
ADD/ADHD	0 (0.0)	0 (0.0)	---	---
Anticoagulant Therapy	19 (47.5)	31 (22.1)	0.002	3.18 (1.52, 6.65)
Angina Pectoris	0 (0.0)	0 (0.0)	---	---
Mental/Personality Disorder	0 (0.0)	2 (1.4)	0.447	---
Myocardial Infarction	1 (2.5)	1 (0.7)	0.342	3.56 (0.21, 58.28)
Peripheral Arterial Disease	3 (7.5)	2 (1.4)	0.039	5.59 (0.90, 34.72)
Substance Abuse Disorder	3 (7.5)	2 (1.4)	0.039	5.59 (0.90, 34.72)
At least one morbidity incident	15 (37.5)	22 (15.7)	0.003	3.21 (1.46, 7.05)
Mortality	3 (7.5)	0 (0.0)	0.001	---

**Table 6:** Post-intervention patients “at risk” for ICU admission with risk factors of CCI, OFS, ED vitals, BMI, Hb/HCT, INR, P/F ratio, LOS, and transfusion products Abbreviations: ICU; intensive care unit; CCI, Charlson comorbidity index; OFS, orthopedic frailty score; ED, emergency department; BMI, body mass index; INR, international normalized ratio; LOS, length of stay; SD, standard deviation; n, sample size; HR, heart rate; bpm, beats per minute; RR, respiratory rate; SBP, systolic blood pressure; DBP, diastolic blood pressure; P/F ratio, partial pressure of oxygen in arterial blood (PaO2) to the fraction of inspiratory oxygen concentration (FiO2); GCS, Glasgow coma score; ISS, injury severity score; ---, not applicable.

Mean ± SD	Admitted to ICU (n=40)	Not Admitted to ICU (n=140)	ρ value	95% CI
Age	81.9 ± 9.7	82.4 ± 8.9	0.725	81.00, 83.69
CCI Score	1.1 ± 1.1	0.8 ± 1.1	0.209	0.76, 1.09
Hip Frailty Score	1.1 ± 0.8	1.2 ± 0.9	0.494	1.04, 1.30
Number of comorbidities	3.1 ± 1.5	2.5 ± 1.4	0.034	2.49, 2.91
Initial ED HR (bpm)	82.1 ± 17.8	79.0 ± 15.3	0.279	77.36, 82.07
Initial ED RR, breaths per minute	19.9 ± 3.8	18.3 ± 2.1	0.022	18.31, 19.09
Initial ED SBP (mmHg)	139.6 ± 27.4	149.0 ± 26.3	0.055	143.00, 150.96
Initial DBP (mmHg)	69.5 ± 18.8	74.0 ± 16.9	0.157	70.45, 75.62
Initial ED O2 Sat	96.0 ± 3.4	96.7 ± 2.7	0.183	96.15, 97.00
Revised Trauma Score	7.7 ± 0.6	7.8 ± 0.2	0.257	7.74, 7.85
Body Measure Index (BMI)	23.5 ± 4.8	24.5 ± 5.1	0.278	23.56, 25.04
Hematocrit (HCT)	35.8 ± 6.3	37.4 ± 5.4	0.101	36.28, 37.93
INR	1.2 ± 0.5	1.1 ± 0.2	0.099	1.09, 1.19
Lactate	1.8 ± 1.0	1.6 ± 0.7	0.415	1.52, 1.96
Base Excess or Base Deficit (BE/BD)	1.6 ± 1.8	5.1 ± 6.4	0.149	0.96, 5.60
P/F Ratio	334.00 ± 126.3	387.1 ± 172.2	0.466	286.45, 434.65
GCS	14.4 ± 2.0	14.8 ± 0.7	0.282	14.55, 14.90
ISS	10.4 ± 2.8	9.4 ± 1.2	0.030	9.39, 9.90
Hospital LOS (days)	8.1 ± 5.9	5.2 ± 2.0	0.003	5.34, 6.37
ICU LOS (days)	4.7 ± 4.6	---	---	3.28, 6.21
Total Vent Days	2.0 ± 1.0	---	---	0.00, 4.48
PRBC 0-24 hrs	1.5 ± 0.8	1.1 ± 1.0	0.153	1.05, 1.66
Plasma 0-24 hrs	0.6 ± 1.2	0.2 ± 1.0	0.460	0.00, 0.85
Platelets 0-24 hrs	0.0 ± 0.0	0.0 ± 0.0	---	---
Cryo 0-24 hrs	0.0 ± 0.0	0.0 ± 0.0	---	---
VLLA 0-24 hrs	0.0 ± 0.0	0.0 ± 0.0	---	---
Cellsaver 0-24 hrs	0.0 ± 0.0	0.0 ± 0.0	---	---

**Table 7:** Charge for intervention Total cost per patient for intervention = $282.12. Abbreviations: HC, hospital charge; CBC, complete blood count; PLT, platelets; COMP, comprehensive; B-TYPE, brain; I, inhibitory character; T, tropomyosin; DX, diagnosis.

Charge Code - Charge Code Description	Direct Unit Cost
HC CBC PLT automated	$7.70
HC Comp metabolic panel	$15.91
HC lactic acid	$8.72
HC B-type natriuretic peptide	$24.31
HC troponin I T	$8.44
HC DX chest 1 view	$92.04

## Discussion

Hip fracture patients have high morbidity and mortality due to multiple risk factors, including age, comorbidities, frailty, and surgical urgency. Strategies utilized to reduce hip fracture morbidity and mortality include semi-emergent surgery within 24 hours, ortho-geriatric consultation, and ICU admission for monitoring and care. To address these factors, a screening protocol was developed and implemented for the evaluation and postoperative screening of hip fracture patients to determine the need for planned ICU admission. This protocol was implemented to improve our clinical outcomes, including mortality for hip fracture patients at our institution. The Trauma Quality Improvement Program (TQIP) is an American College of Surgeons (ACS)-validated national benchmarking dataset, with 900 participating trauma centers. Our TQIP percentile score for hip fracture patients compared to similar US hospitals was an outlier with relatively higher mortality.

The screening process developed incorporated validated assessments for identifying patients who are at high risk of requiring an ICU admission. The clinical examination included Frailty index, presenting vital signs and oxygen saturation, GCS, ISS, and body mass index (BMI). Postoperative laboratory tests included BNP, troponin, lactate, and routine tests such as CBC and CMP. Additional testing included chest radiograph and electrocardiography. Clinical nomograms have been developed, which can accurately predict unplanned ICU admission after hip fracture surgery. Various independent risk factors identified include age, chronic heart failure, coronary artery disease, chronic pulmonary disease, Parkinson's disease, serum albumin, and creatinine concentrations.

Our results showed that, of the 470 patients (over a 1.0-year period) included in the study, 40 patients were admitted to the ICU (8.5%) post-intervention. Twenty-five of these patients were observed overnight and remained stable with minimal intervention needed. However, 15 patients admitted to the ICU deteriorated clinically validating their need for critical care monitoring and admission to the ICU. When patient samples were analyzed using only PS-matched groups, there was a significant decrease (p=0.45) in unplanned ICU admissions after screening protocol implementation (Table 4). Significant risk factors for ICU admission included ED RR, ISS, number of comorbidities, SAD, PAD, anticoagulant use, and COPD.

Some of these risk factors have been identified in prior studies. ED tachypnea is a signal for acute respiratory failure, which correlates with known risk factors for ICU admission, such as pulmonary or cardiac disease [15,19-24]. ISS or increasing injury severity is a known risk factor for ICU admission, which can lead to increased incidence of post-injury sepsis or multiple organ failure [10]. The number of comorbidities is indicative of pulmonary, renal, hepatic, and cardiac disease. These are known risk factors for ICU admission [20-22]. SAD may reflect the risk associated with withdrawal syndromes and metabolic derangements caused by these substances. Anticoagulant use may increase bleeding risk and contribute to acute blood loss anemia and peri-operative hypotension in hip fracture patients, which are known risk factors for ICU admission [15,23]. In our institution, it is rare to reverse coagulopathy due to anticoagulants for hip fracture patients, except those on coumadin with a high INR. The PAUSE study revealed elective cases on DOAC drugs could safely have their drugs held preoperatively and restarted postoperatively without the requirement for heparin bridging or reversal [25]. COPD is a subset of pulmonary disease that has been associated with increase risk of ICU admission [15,21,22]. This is most likely due to the need for supplemental oxygen in the form of high-flow nasal cannula, ventilatory support with non-invasive positive pressure ventilation (NIPPV), or endotracheal intubation and ventilatory support. There is a significant association between PAD and hip fractures [26]. Our study also suggests that PAD is a risk factor for ICU admission in hip fracture patients.

The statistically significant reduction in unplanned ICU admission and increase in planned ICU admission in our study has been shown to reduce mortality in prior studies [23,24].

Although mortality was not significantly reduced in pre- and post-intervention groups, 30-day mortality reduced from 4.8% in 2021 to 2.5% in 2022 and 2.7% in 2023 (ytd). This is well below the 10% national mortality metric for hip fracture registries. Due to the small sample size in the post-intervention group, it is difficult to say if this will translate consistently into improved mortality in the future.

However, we achieved our TQIP goal of improving our hip fracture outcomes. Our hospital-specific new TQIP data for 2023 confirmed improvement in clinical outcomes. We changed from a high outlier with respect to hospital mortality in hip fracture patients in our 2022 TQIP report to a medium-tier hospital with comparable mortality to other similar benchmarked hospitals. A multi-institutional prospective randomized study comparing usual care with the new screening protocol may be helpful in providing clarity to this clinical question.

However, the study did have limitations. These included a non-randomized single-site retrospective design, a small post-intervention sample admitted to the ICU, and missing data on some variables. The methodological limitation of a non-randomized study was addressed in the analysis when only PS-matched groups were compared. The small post-intervention sample admitted to the ICU could not be controlled for; however, 95% CIs were reported to demonstrate the influence of a small sample size on point estimates. Missing data were not addressed with imputations as there was less than 5% missing data. Many studies also use a geriatric age of 65 years for evaluating hip fracture patients. We used 55 years of age as our geriatric cut-off. Some studies have shown an increased risk of mortality in trauma patients above this age. We had only a few patients in this 55-65 age range, which did not affect our statistical analysis.

As the world population of older adults, in particular those over age 85, increases, the incidence of fragility fractures is expected to also increase. It is predicted that the worldwide incidence of hip fractures will grow to 6.3 million annually by 2050 [1]. Fractures result in significant financial and personal costs. These geriatric patients are at risk for significant functional decline and mortality. Various risk stratification strategies have been adopted to select perioperative hip fracture patients for ICU care, including RSI [15] and nomograms [19].

## Conclusions

Though this retrospective study contained a relatively small sample size, this postoperative multi-modality screening intervention encompassing clinical examination, laboratory values, presenting vital signs, radiologic images, and EKG has a low financial impact and is universally applicable in similar trauma centers. The interventional screening protocol provides data to support hospital allocation decisions of scarce critical care resources for hip fracture patients who require and would benefit most from a higher level of care. Screening postoperative hip fracture patients for ICU admission is an easily reproducible, low-cost method to make the most strategic use of limited resources and reduce unplanned ICU admissions when treating this subset of high-risk trauma patients. Replication of this screening intervention with a larger sample could yield further information on the effectiveness and benefits derived from this multi-modality protocol.
